# Renal safety of postoperative short-term use of flurbiprofen axetil in well-controlled hypertensive patients: a prospective multicenter cohort study

**DOI:** 10.3389/fphar.2026.1773939

**Published:** 2026-04-22

**Authors:** Liang Sun, Yuhui Jiang, Xiaoran Ma, Yanhong Liu, Yubo Xie, Xiaoxia An, Weidong Mi, Yi Feng

**Affiliations:** 1 Department of Anesthesiology, Peking University People’s Hospital, Beijing, China; 2 Department of Anesthesiology, Peking University People’s Hospital Shijiazhuang Campus/Shijiazhuang People’s Hospital, Shijiazhang, China; 3 Department of Anesthesiology, 1st Medical Center of Chinese PLA General Hospital, Beijing, China; 4 Department of Anesthesiology, The First Affiliated Hospital of Guangxi Medical University, Nanning, China; 5 Department of Anesthesiology, The First Affiliated Hospital of Zhejiang University School of Medicine, Hangzhou, China

**Keywords:** acute kidney injury, flurbiprofen axetil, renal safety, total knee arthroplasty, tubular damage, well-controlled hypertension

## Abstract

**Background:**

Currently, postoperative nonsteroidal anti-inflammatory drugs-associated kidney damage in hypertensive patients remains undetermined. This study aimed to examine renal safety of short-term post-surgery use of flurbiprofen axetil (FA) in well-controlled hypertensive patients.

**Methods:**

Data were collected from January 2020 to March 2021 at four major hospitals on patients who had undergone total knee replacement surgery (TKA). Patients were divided into well-controlled hypertensive and normotensive groups, and received short-term intravenous FA postoperatively. The primary outcomes were trajectory changes of postoperative renal injury biomarkers. Second outcomes included acute kidney injury (AKI), pain intensity and other complications.

**Results:**

A total of 120 patients were enrolled. Post-surgery FA significantly reduced pain in both groups. The results of repeated measures analysis of variance (ANOVA) demonstrated that the main effects of group, time and the group-by-time interaction on blood urea nitrogen (BUN), serum creatine (SCr) and Cystatin C (Cys C) levels were not statistically significant on postoperative day 2 and 5 (POD2 and POD5) (all P > 0.05). Urine N-acetyl-beta-glucosaminidase (NAG) levels were significantly higher on POD2 and POD5 in both groups [preoperative: (6.66 ± 4.87) U/L, POD2: (10.07 ± 6.88) U/L, POD5: (10.25 ± 7.68) U/L in normotensive group; preoperative: (7.39 ± 4.07) U/L, POD2: (12.34 ± 7.1) U/L, POD5: (15.57 ± 12.34) U/L in well-controlled hypertensive group; all P < 0.001] compared with baseline. However, only the well-controlled hypertensive group showed a continued increase in NAG levels on POD5 (P = 0.039). AKI occurred in 7.0% (4/57) and 3.2% (2/63) of well-controlled hypertensive and normotensive patients (P = 0.335), and all the AKI patients were at stage 1. After adjusting for confounding factors, hypertension had no impact on AKI (OR = 0.733, 95%CI: 0.074–7.222, P = 0.790). No significant differences in other complications were observed between the groups.

**Conclusion:**

The current FA regimen after surgery is effective, and it does not increase AKI incidence in well-controlled hypertensive TKA patients, thought posing a risk of tubular damage.

## Introduction

Total knee arthroplasty (TKA) stands as the predominant approach to address severe knee functional impairment, notably alleviating pain and enhancing patients’ quality of life (Lenguerrand et al., 2023; Yap et al., 2022). With over a million TKA procedures conducted annually worldwide, this number is anticipated to rise due to population aging (Gasbjerg et al., 2022). Post-TKA, about 30% of patients experience moderate pain, while 60% endure severe pain, which, when improperly managed, can prolong hospitalization and impede functional recovery (Sonawane et al., 2021). Hence, effective postoperative pain control is paramount for optimal outcomes.

To enhance postoperative pain management in TKA, current guidelines advocate for a multimodal analgesia (MMA) strategy, emphasizing reduced opioid usage, particularly within enhanced recovery after surgery (ERAS) protocols (Lubis et al., 2021; Frassanito et al., 2020). Common practices involve nerve blocks such as femoral and sciatic nerve blocks combined with nonsteroidal anti-inflammatory drugs (NSAIDs), which not only effectively alleviate pain but also facilitate improved rehabilitation (Berkowitz et al., 2021).

More is known that NSAIDs exert anti-inflammatory, analgesic, and antipyretic effects by inhibiting cyclooxygenase (COX) enzymes, which in turn suppress prostaglandin and thromboxane synthesis (Tang et al., 2021; Katsuno et al., 2021). Prostaglandins in the kidney act as vasodilators, ensuring sufficient blood flow to the organ. The association between the risk of acute kidney injury (AKI) and chronic NSAID use has been validated even in patients with preserved renal function (Tang et al., 2021), with this risk further heightened in those with existing comorbidities such as hypertension, diabetes mellitus, and hypoalbuminemia (Inaba et al., 2019). However, there is growing concern among clinicians regarding whether perioperative NSAID administration leads to AKI, considered a major complication and safety issue (Yu and Feng, 2021).

Our previous study revealed that clinically relevant medium-dose flurbiprofen axetil (FA, 25 mg/kg/d), an injectable nonselective COX inhibitor, can effectively alleviate postoperative pain and achieve a ceiling effect in spontaneously hypertensive (SH) rats. However, FA decreased glomerular and tubular function in a dose-dependent manner both in normotensive and hypertensive rats. Meanwhile, pathologically renal impairment is detectable earlier after surgery in hypertensive rats, even at the clinically relevant low-dose FA (Sun et al., 2025). FA is not recommended for those with uncontrolled hypertension (i.e., systolic blood pressure >180 mmHg and/or diastolic blood pressure >110 mmHg) in its directions for use. However, conclusive evidence regarding the renal safety of short-term perioperative FA use among well-controlled hypertensive patients is lacking. Considering that blood pressure of TKA patients with hypertension should be controlled well before surgical operation, the primary aim of this study was to determine trajectory changes of renal injury biomarkers when postoperative continuous FA, as part of multimodal analgesia (MMA) protocols were administered in well-controlled hypertensive versus normotensive subjects undergoing primary TKA. A secondary objective was to assess the efficacy of FA in reducing postoperative pain and its impact on postoperative acute kidney injury (AKI) and other related complications.

## Methods

### Study population

This multicenter prospective cohort study was conducted at four tertiary hospitals (Peking University People’s Hospital, Chinese PLA General Hospital, the First Affiliated Hospital of Zhejiang University, and the First Affiliated Hospital of Guangxi Medical University) between January 2020 and March 2021. Consecutively enrolled patients scheduled for TKA were included. The study was approved by the Institutional Review Board of Peking University People’s Hospital (2018PHB043-02), and approval from each institutional review board was obtained according to individual hospital protocols. The study was registered in the Chinese Clinical Trial Registry (http://www.chictr.org.cn/index.aspx) with registration number ChiCTR1800014464. Informed consent was obtained from all patients.

### Inclusion and exclusion criteria

Patients meeting the following inclusion criteria were included: (1) undergoing unilateral TKA surgery under spinal anesthesia; (2) having an American Society of Anesthesiologists (ASA) status of I ∼ III; (3) having either normotension or well-controlled hypertension (clinic systolic/diastolic blood pressure threshold: 140/90 mmHg or home/ambulatory systolic/diastolic blood pressure threshold: 135/85 mmHg, which were being documented by primary care or secondary care in the last 12 months) (Mccormack et al., 2026).

The exclusion criteria were as follows: (1) known allergy to any of the study medications, including FA; (2) impaired consciousness or cognitive dysfunction; (3) chronic kidney disease according to the Kidney Disease Improving Global Outcome (KIDGO) criteria (Patel et al., 2024); (4) failure to provide informed consent.

### Study protocol

Patients previously diagnosed with hypertension were categorized into the well-controlled hypertensive group, while those with no history of hypertension were placed in the normotensive group. Preoperative antihypertensive medications were continued on the day of surgery, which aligns with international recommendations, except for angiotensin-converting enzyme inhibitors (ACEIs) and angiotensin receptor blockers (ARBs), which may cause hypotension during surgery (Thompson et al., 2024).

All TKA surgeries were performed under spinal anesthesia using 10–15 mg of bupivacaine. Intraoperative sedation was determined based on the preference of the anesthesiologist in the study.

A medial parapatellar approach was utilized for TKA, with all prostheses being posterior-stabilized and cemented, and the patella was resurfaced. A tourniquet was applied in all cases and released after wound closure. A deep drain was inserted for drainage and remained in the knee for 24 h. Intravenous tranexamic acid (1 g) was administered to patients 15 min before the skin incision to reduce postoperative bleeding. Intraoperative mean arterial pressure (MAP) values were maintained at least 65 mmHg. Usual care (fluid infusion, vasoactive drugs etc.) were initiated to avoid sustained MAP values lower than 65 mmHg, which were consistent with current standards of practice and the European Society of Cardiology guideline recommendations (Kristensen et al., 2014).

Postoperatively, analgesic procedures were carried out by the anesthesiologist using a multimodal strategy, comprising two methods: (1) intravenous analgesic protocol: a loading dose of 100 mg FA was administered intravenously 30 min before the end of the operation, followed by continuous intravenous infusion of FA via a mechanical pump at a rate of 4 mg/h [formula: FA 40 mL (400 mg) + 0.9% sodium chloride 60 mL, total volume 100 mL] until its depletion (lasting for 50 h). (2) ultrasound-guided peripheral nerve block protocol: a single sciatic nerve block was performed using 0.15% ropivacaine (20 mL), once the femoral catheter was sited, continuous femoral block was initiated with a loading dose of 0.3% ropivacaine (20 mL), followed by patient-controlled nerve analgesia (PCNA) using 0.2% ropivacaine through an electronic pump (background dose: 5 mL/h, bolus dose: 10 mL, lockout time: 60 min, total volume: 300 mL).

Early ambulation and anticoagulation were standard prophylactic measures against deep venous thrombosis postoperatively. Pain intensity was assessed by a nurse using a numeric rating scale (NRS) ranging from 0 (no pain) to 10 (extreme pain) on postoperative days 1 (POD 1) and 2 (POD 2). Intravenous tramadol (1 mg/kg) or morphine (0.1 mg/kg) was administered as rescue analgesia if the NRS score was ≥4.

Blood samples were collected at 8:00 a.m. before surgery and on POD 2 and POD5, then centrifuged (2,000 g for 15 min), and the serum was separated and stored at −80 °C for analysis. Additionally, 10 mL of urine from each patient at the specified time points were collected.

### Data collection

Baseline covariates were gathered to account for a range of clinically relevant factors. Preoperative parameters included gender, age, height, weight, body mass index (BMI), diabetes mellitus, midazolam medication, level of hemoglobin, incidence of hypoalbuminemia, and ASA grade. Intraoperative variables encompassed surgery duration, occurrences of hypotension, volume of fluid therapy, urine output, blood loss, use of cell saver, and allogeneic transfusion. Postoperative data included oliguria (urine output <0.5 mL/kg/h or <0.3 mL/kg/h) according to diagnosing for staging of AKI (Mishra et al., 2022; Bianchi et al., 2021), allogeneic transfusion, numeric rating scale (NRS) score for pain assessment, analgesia-related complications (such as postoperative nausea and vomiting (PONV), dizziness, and gastrointestinal bleeding), and frequency of rescue analgesic requirements.

### Measurements for renal injury biomarkers

Serum levels of sodium (Na^+^), potassium (K^+^), blood urea nitrogen (BUN), serum creatinine (SCr), cystatin C (Cys c), and urine N-acetyl-beta-glucosaminidase (NAG) activity were determined using standard autoanalyzer techniques with a Rayto automatic biochemical analyzer in the clinical laboratory of Peking University People’s Hospital.

### Incidence and severity of AKI

Postoperative AKI was diagnosed on the basis of changes in SCr within 7 days after surgery according to the KDIGO SCr-based criteria: either an increase in SCr by ≥ 0.3 mg/dL (26.5 μmol/L) within 48 h or an increase in SCr to ≥1.5 times the baseline within 7 days at any time during follow-up (Tang et al., 2021). Severity of AKI were also evaluated according to KDIGO criteria (Mishra et al., 2022). Baseline SCr was defined as the closest SCr measurement to the surgery day, obtained after admission to the hospital and before the surgery. In cases where multiple AKI events occurred, the earliest event was considered the outcome.

### Statistical analysis

Based on preliminary observations, Cys C levels on POD2 were (1.03 ± 0.15) mg/L for well-controlled hypertensive patients and (0.91 ± 0.15) mg/L for normotensive patients. Using PASS software (Power Analysis and Sample Size 14.0, NCSS, LLC., Kaysville, UT, United States), a sample size of 44 patients per group was calculated with α = 0.05 and β = 0.1, assuming equal group sizes. Accounting for a 20% dropout rate, 55 patients per group were deemed necessary.

Data were presented as mean ± standard deviation (SD) for normally distributed continuous variables or median [interquartile range] for skewed distributions, while categorical variables were expressed as frequencies or percentages. Normal distribution was assessed using the Kolmogorov-Smirnov test. Independent sample t-tests or Mann-Whitney U tests were used for between-group comparisons of continuous variables, and paired t-tests were employed for within-group comparisons. The chi-square test was utilized for pairwise comparison of categorical data. For the comparison of repeated measures data, a repeated measures analysis of variance (ANOVA) was performed. If Mauchly’s test of sphericity indicates a violation of the sphericity assumption (P < 0.05), an epsilon (ε) correction is required, and the Greenhouse-Geisser correction method was used for data analysis. Multivariate logistic regression analysis was conducted to assess the influence of hypertension on AKI while controlling for relevant covariates. Statistical analysis was performed using SPSS 19.0 software package, with a significance level set at P < 0.05.

## Results

### Demographic and clinical characteristics among well-controlled hypertensive versus normotensive patients

**FIGURE 1:**
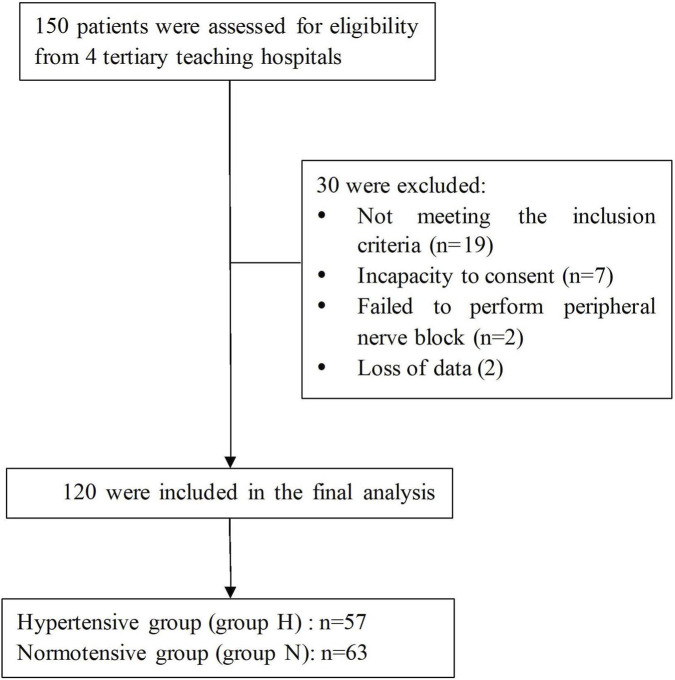
Flowchart of the study.

A total of 120 patients were included in the study, comprising 57 cases in the well-controlled hypertensive group and 63 cases in the normotensive group, as depicted in Figure 1. Detailed perioperative demographic and clinical data, including preoperative, intraoperative, and postoperative variables, are presented in Table 1. There were statistically significant differences in age (P = 0.003), weight (P < 0.001), body mass index (BMI) (P < 0.001), and ASA grade (P < 0.001) between the well-controlled hypertensive and normotensive groups. However, no significant differences were observed between the two groups regarding other variables.

**TABLE 1:** Demographic and clinical characteristics of patients.

Parameters	Group H	group N	t/χ^2^/Z	P Value
	(n = 57)	(n = 63)		
Preoperative				
Male, n (%)	11 (19.3%)	10 (15.9%)	0.243	0.622
Age (years)	67.0 ± 6.0	63.3 ± 7.3	3.021	0.003
Height (cm)	158.9 ± 7.7	158.2 ± 6.6	0.588	0.558
Weight (kg)	69.7 ± 10.0	62.0 ± 12.0	3.780	<0.001
BMI (kg/m^2^)	27.6 ± 3.1	24.8 ± 4.4	3.967	<0.001
Diabetes n (%)	13 (22.8%)	8 (12.7%)	2.118	0.146
Hypoalbuminemia n (%)	24 (42.1%)	21 (33.3)	0.982	0.322
ASA grade	​	​	15.495	<0.001
I n (%)	0 (%)	13 (20.6%)	​	​
II n (%)	52 (91.2%)	49 (77.8%)	​	​
III n (%)	5 (8.8%)	1 (1.6%)	​	​
Intraoperative				
Surgery time (min)	160.4 ± 44.9	152.6 ± 39.7	1.011	0.314
Hypotension n (%)	20 (35.1%)	21 (33.3%)	0.041	0.840
Crystal (mL)	1077 ± 425	1047 ± 452	0.378	0.706
Colloid (mL)^a^	0 [0–500]	0 [0–500]	0.505	0.613
Urine output (mL)^a^	200 [0–500]	200 [0–400]	0.244	0.807
Blood loss (mL)^a^	100 [50–200]	80 [50–150]	1.150	0.250
Net fluid input (mL)	939 ± 385	959 ± 320	−0.311	0.757
Cell saver n (%)	9 (15.8%)	5 (7.9%)	1.791	0.181
Cell saver (mL)	0 [0–0]	0 [0–0]	1.293	0.196
Allogenic transfusion n (%)	5 (8.8%)	7 (11.1%)	0.182	0.670
Packed red blood cell transfusion (mL)^a^	0 [0–0]	0 [0–0]	−0.419	0.675
Postoperative				
Allogenic transfusion n (%)	0	0	—	—
Oliguria n (%)	0	0	—	—

Values are presented as the mean ± standard deviations for normally distributed continuous variables or median [interquartile range] if distributions were skewed, while categorical variables were expressed as frequencies or percentages. Independent sample t-test or Mann-Whitney U test (a) was performed between the groups as appropriate. BMI = body mass index; ASA = american society of anesthesiologists physical status.

### Postoperative NRS at rest and movement and rescue analgesia

**FIGURE 2:**
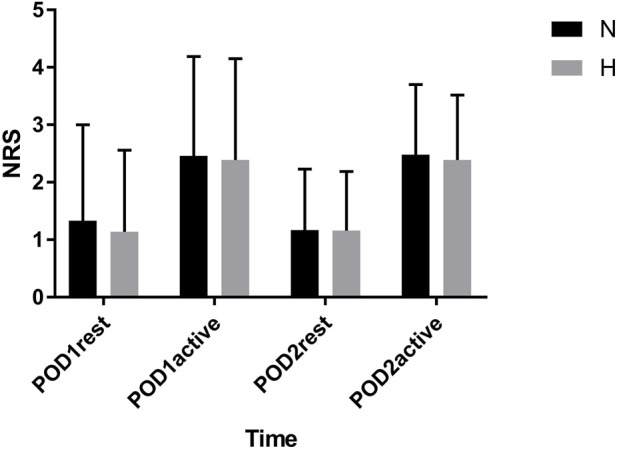
NRS score on POD1 and POD2. NRS = numeric rating scale; POD = postoperative day.

The pain intensity of patients in both groups at rest and during movement on POD1 and POD2 was illustrated in Figure 2. There were no statistically significant differences in NRS scores between the two groups at different time points and under different conditions (all P > 0.05). The incidence of rescue analgesia in the well-controlled hypertensive group and normotensive group was 5.3% and 6.3%, respectively, with no statistically significant difference (P = 0.8) (Table 2).

**TABLE 2:** Postoperative complications and rescue analgesia.

Parameters	Group H	group N	χ^2^/*U*	P Value
	(n = 57)	(n = 63)		
AKI n (%)	4 (7.0%)	2 (3.2%)	0.930	0.335
AKI stage n (%)	​	​	4.000	1.000
1	4 (7.0%)	2 (3.2%)	​	​
2	0	0	​	​
3	0	0	​	​
Dizziness on POD1 (%)	3 (5.3%)	7 (11.1%)	1.340	0.247
Dizziness on POD2 (%)	2 (3.5%)	2 (3.2%)	0.010	0.919
PONV on POD1 (%)	5 (8.8%)	11 (17.5%)	1.955	0.162
PONV on POD2 (%)	2 (3.5%)	3 (4.8%)	0.118	0.732
Gastrointestinal bleeding n	0	0	—	—
Rescue analgesia (%)	3 (5.3%)	4 (6.3%)	0.064	0.800

Values are presented as the number (%). Data were analyzed using the Chi-squared test or Mann–Whitney U test. AKI = acute kidney injury; POD = postoperative day; PONV = postoperative nausea and vomiting.

### Serum sodium and potassium levels

The results of repeated measures ANOVA showed that the main effects of group and the group-by-time interaction on serum Na^+^ levels were not statistically significant (F = 1.199, P = 0.276; F = 0.478, P = 0.598), however, a statistically significant difference was observed in the main effect of time (F = 3.943, P = 0.026).

**FIGURE 3:**
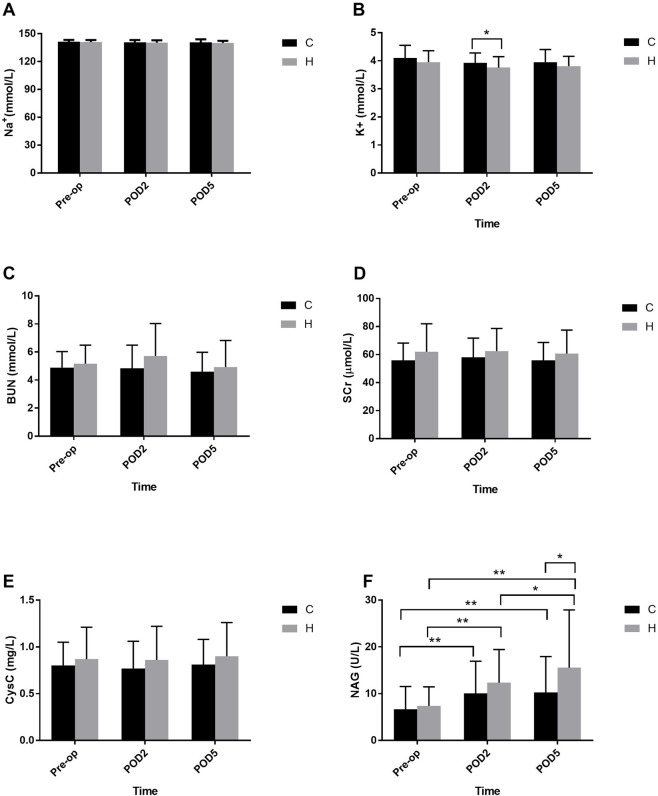
Trajectory changes of serum sodium **(A)** and potassium **(B)**, BUN **(C)**, SCr **(D)**, Cys C **(E)**, and NAG activity **(F)** before surgery, on POD2 and POD5. BUN = blood urea nitrogen; SCr = serum creatine; Cys C = cystatin C; NAG = urine N-acetyl-beta-glucosaminidase; POD = postoperative day. *P < 0.05, **P < 0.01.

As for the serum K^+^ levels, the main effects of group and time were statistically significant (F = 6.468, P = 0.013; F = 9.401, P < 0.001), however, there was no statistically significant difference in the group-by-time interaction effect (F = 0.059, P = 0.930), although a statistically significant difference was detected in serum K^+^ on POD2 (P = 0.026). Nevertheless, serum Na^+^ and K^+^ levels were all within the clinical normal range (Figures 3A,B).

### Traditional renal injury biomarkers

#### Serum BUN

The BUN levels of both groups before surgery, on POD2, and POD5 were illustrated in Figure 3C. Repeated measures ANOVA showed that the main effects of group, time and the group-by-time interaction on BUN were all not statistically significant (F = 2.508, P = 0.116; F = 0.397, P = 0.541; F = 0.905, P = 0.349).

#### Serum creatinine

The SCr levels before surgery, on POD2, and POD5 were all within the normal range (Figure 3D). Similar to serum BUN, the results of repeated measures ANOVA demonstrated that the main effects of group, time and the group-by-time interaction on SCr were not statistically significant (F = 1.206, P = 0.274; F = 0.995, P = 0.321; F = 1.032, P = 0.312).

### Novel renal injury biomarkers

#### Serum cystatin C

The Cys C levels of both patient groups before operation, on POD2, and POD5 were within the normal clinical range (Figure 3E). Likewise, repeated measures ANOVA failed to detect statistically significant differences in the main effects of group, time and the group-by-time interaction on Cys C (F = 0.082, P = 0.775; F = 1.272, P = 0.268; F = 0.936, P = 0.348).

#### Urinary NAG activity

Repeated measures ANOVA observed that the main effects of group, time and the group-by-time interaction on NAG were statistically significant (F = 7.613, P = 0.007; F = 26.208, P < 0.001; F = 3.737, P = 0.031). Compared with baseline, the urinary NAG activity of both groups on POD2 and POD5 was significantly higher [preoperative: (6.66 ± 4.87) U/L, POD2: (10.07 ± 6.88) U/L, POD5: (10.25 ± 7.68) U/L in normotensive group; preoperative: (7.39 ± 4.07) U/L, POD2: (12.34 ± 7.1) U/L, POD5: (15.57 ± 12.34) U/L in well-controlled hypertensive group; all P < 0.001]. Moreover, the level of NAG in the normotensive group did not continue to increase on POD5 (P > 0.05). However, the NAG level in the well-controlled hypertensive group continued to increase (P = 0.039) and exceeded the upper limit of the normal range on POD5 compared with that of POD2 (Figure 3F).

#### Incidence and severity of AKI

Among the 120 patients, 6 (5%) developed AKI as defined by the KDIGO criteria. The incidence of AKI in the well-controlled hypertensive group and normotensive group was 7.0% (4/57) and 3.2% (2/63), respectively, but there was no statistically significant difference (P = 0.335). Moreover, all the AKI patients were at stage 1, and no statistically significant difference of AKI severity between the two groups was observed (P = 1.000). After adjusting for age, weight, BMI, and ASA grade, multivariate logistic regression analysis revealed that hypertension had no impact on AKI (OR = 0.733, 95%CI: 0.074–7.222, P = 0.790) (Table 2).

#### Incidence of other complications

Regarding analgesia-related adverse effects, there were no significant differences in the incidence of PONV, dizziness and gastrointestinal bleeding postoperatively (all P > 0.05) (Table 2).

## Discussion

The present study indicated that the average pain scores (NRS) of both groups at rest and during movement on POD1 and POD2 were below 3, suggesting that the current postoperative FA regimen, combined with PCNA as part of MMA, effectively provides pain relief in patients undergoing TKA. As the population ages, hypertension has become a common condition among TKA patients. As mentioned earlier, hypertension can increase the risk of renal injury when NSAIDs are administered (Inaba et al., 2019). However, during postoperative follow-up, we observed no difference in the incidence of AKI between the two group, which suggests that our MMA regimen, utilizing an FA pump in TKA patients, does not increase the risk of AKI in well-controlled hypertensive patients.

Urine output, BUN, and SCr are currently the primary indicators for clinically evaluating renal function. Despite being easy to monitor, they may still fall within the normal range when GFR is slightly decreased due to the strong compensatory function of the kidney. Consequently, they cannot reflect early or mild renal function damage.

Cys C, a non-glycosylated protein composed of 120 amino acids, is an alkaline secretory substance whose levels remain unaffected by age, sex, diet, inflammation, tumors, drugs, or muscle content. It serves as an early diagnostic marker for glomerular filtration and is a sensitive biological indicator of impaired function in various conditions including transplantation, corticosteroid use, disorders associated with reduced muscle mass or cachexia, malignancy, and obesity (Hu et al., 2017; Teaford et al., 2020). However, our study did not find any differences in Cys C levels at different time points between the two groups, and a notable variance exists between these results and our earlier animal experiment findings. We ascribe this variance partly stems from the subtypes of hypertension: patients enrolled in this cohort study were well-controlled hypertensive subjects. Moreover, a previous study also reported that Cys C failed to detect AKI in the early postoperative period following cardiac surgery (Mutlu et al., 2020). Another study involving 150 patients even found that Cys C levels measured at the 2nd postoperative hour were lower than preoperative levels in the AKI group (Wald et al., 2010). Therefore, clear evidence on this topic is lacking and further investigation is warranted.

NAG, or N-acetyl-β-D-glucosaminidase, is primarily found in the cell lysosomes of the brush border of the proximal tubule. When renal tubular epithelial cells are damaged and lysosomal enzymes overflow, urine NAG activity increases significantly. It serves as a sensitive and reliable indicator reflecting damage to renal tubular epithelial cells (D'Amico and Bazzi, 2003; Skálová, 2005). It has been reported that FA can induce acute tubulointerstitial nephritis (Kaufhold et al., 1991) and tubular damage (Sun et al., 2025). Similarly, this study observed that FA can exert damaging effects on the renal tubules in patients with hypertension, but not in normotensive patients. This suggests that tubular injury should be monitored when NSAIDs are prescribed during the perioperative period, especially at high doses.

The link between perioperative NSAIDs and postoperative AKI is still debated due to past studies, particularly concerning individuals with preexisting health issues. Our study found that well-controlled hypertension did not pose an additional risk in TKA patients using short-term FA (200 mg/d) in terms of AKI after adjusting for relevant important influencing factors, though traditional (BUN) and novel sensitive (NAG) kidney injury indicators experienced a transient increase during the postoperative period. In fact, it makes sense, minor physical damage does not always lead to considerable dysfunction of certain organs. A previous large single-center retrospective study revealed FA’s effect on postoperative AKI was dose-dependent, and using a low dose of FA (50–100 mg) perioperatively was even significantly associated with a decreased incidence of postoperative AKI (Wang et al., 2020). However, when the dose reached ≥250 mg, FA significantly increased the incidence of AKI, but the medium dose of FA (100–250 mg) had no impact on AKI. Likewise, a different retrospective analysis of 9246 patients who received perioperative parecoxib (a selective COX-2 inhibitor) showed a slight decrease in postoperative AKI risk among adult patients undergoing non-cardiac surgery (Tang et al., 2021). This finding was opposed to the traditional concept: NSAIDs were an important cause of AKI (Jeon et al., 2021).

The underlying mechanisms responsible for the varying biological effects on renal function depending on the dose of FA remain unclear. A large dose of FA may inhibit COX, which suppresses the production of prostaglandins, leading to decreased renal blood flow, tubular obstruction, and direct cytotoxicity or cell-mediated immune injury. This can cause renal papillary necrosis and acute tubulointerstitial nephritis (Colomé et al., 1991). Hence, large doses of NSAIDs have been implicated as causes of AKI, especially in the aging population with multiple comorbidities (e.g., hypertension). COX derivatives may play a crucial role in the pathogenesis of progressive nephropathies. The potential role of prostanoids in the pathogenesis of progressive nephropathies has long been recognized. Stimulation of podocytes by complement fractions can increase the local synthesis of prostanoids. Similarly, non-immune mechanisms such as mesangial stretching can increase the expression of COX and enhance the production of its derivatives (Akai et al., 1994). A previous study found that COX-2 could mediate inflammation and structural injury in the glomeruli (Gonçalves et al., 2004). Moreover, part of the renal injury secondary to the operation is attributed to the inflammatory response involving COX-2 and prostaglandins, as well as the production and secretion of chemokines and cytokines. Therefore, a low dose of FA could exert renal protective effects by reducing the inflammatory effects on the kidney (Calistro et al., 2015). Additionally, surgery and pain themselves could activate the renin-angiotensin system (RAS) and increase the secretion of antidiuretic hormone, ultimately leading to hemodynamic abnormalities (Sear, 2005). FA could reduce pain, which may facilitate the restoration of the RAS and renal hemodynamics. Furthermore, FA is highly lipophilic by merging into a lipid microsphere carrier, and may mainly accumulate in the surgical incision and inflammatory site (Zhang et al., 2023), possibly exerting a minor effect on the kidney than other NSAIDs. Certainly, further *in vivo* and *in vitro* studies are warranted to confirm this. Nevertheless, our FA regimen was a moderate dose, and did not increase the risk of postoperative AKI in well-controlled hypertensive patients.

Our study has several limitations. Firstly, AKI was second outcome and determined according to the KDIGO criteria using changes in Scr; however, accurate postoperative urine output data were not collected, but no oliguria case was reported. While most previous studies did not include urine output criteria to diagnose AKI, its inclusion could potentially alter the incidence of AKI. Secondly, the results of this study were obtained from well-controlled hypertensive patients, and it remains unclear whether FA is safe in patients with unstable hypertension. Thirdly, FA dose in this study was a medium clinical dose, further verification is needed to determine whether other doses of FA can ensure analgesic effectiveness without causing additional renal risk in well-controlled hypertensive patients. Fourthly, the challenge of obtaining samples on other postoperative days and post-discharge (after POD 5) hindered our ability to continue monitoring the NAG activity in these patients (most patients were discharged and continued joint rehabilitation training in the rehabilitation center), and the implications of tubular damages in these patients warrants further investigation. Consequently, we were unable to determine the exact time frame for the recovery of renal tubular damage. Finally, there was no statistical difference in the incidence of AKI in this study, but due to the limitation of sample size, challenge of consecutive blood samples on PODs, and the design nature of the cohort study, the renal safety of FA in well-controlled hypertensive patients still needs to be explored in clinical practice by future randomized controlled trials.

## Conclusion

In conclusion, our study demonstrated that postoperative short-term use of FA as part of an MMA regimen effectively reduced pain intensity and did not pose an additional risk of AKI in well-controlled hypertensive patients. However, novel tubular injury biomarker increased during the early postoperative period in those patients, therefore, more future studies are warranted to further explore the renal safety of FA in such patient applications.

## Data Availability

The raw data supporting the conclusions of this article will be made available upon reasonable request to the corresponding author.
